# Physical and mental disability is evident 8 years after diagnosis in early rheumatoid arthritis despite contemporary medication and non-pharmacological interventions

**DOI:** 10.1007/s10067-025-07399-8

**Published:** 2025-04-09

**Authors:** Ingrid Thyberg, Magnus Husberg, Alf Kastbom

**Affiliations:** 1 Department of Biomedical and Clinical Sciences, Division of Inflammation and Infection/Rheumatology, Linköping University, 581 85 Linköping, Sweden; 2 Department of Health, Medicine and Caring Sciences, Linköping University, Linköping, Sweden

**Keywords:** Health Assessment Questionnaire, Patient-reported outcome, Rehabilitation, SF-36

## Abstract

**Introduction:**

Early interventions are known to reduce disease activity and physical disability in rheumatoid arthritis (RA), but less is known about mental health, especially in the era of early active pharmacotherapy. Consequently, we compared long-term physical and mental disability in an early RA cohort (1996–1998) with a later cohort (2006–2008).

**Methods:**

We compared 320 patients from our project Early Intervention in RA (1996–1998) (TIRA-1) with 463 patients from TIRA-2 (2006–2008). During the 8-year follow-up, pharmacotherapy and multi-professional interventions were offered according to guidelines. Disease Activity Score (DAS28), prescribed disease-modifying antirheumatic drugs (DMARDs), Health Assessment Questionnaire (HAQ), and Short Form Health Survey (SF-36) were registered yearly.

**Results:**

Significantly more patients were prescribed DMARDs in TIRA-2 than in TIRA-1, and initial improvements were seen for DAS28 and disability in both cohorts. At follow-up, TIRA-2 patients reported less physical disability (HAQ) and less mental disability (SF-36) than TIRA-1 patients. Despite improvements, 32% of the women and 21% of the men in the TIRA-2 cohort reported considerable disability (HAQ ≥ 1) at the 8-year follow-up.

**Conclusions:**

Despite improvements in our contemporarily treated TIRA-2 cohort, physical and mental disability was evident 8 years after diagnosis, especially among women. These results suggest a forthcoming need for person-centered non-pharmacological rehabilitation programs to optimize physical and mental function and to improve participation in daily life in RA. Also, the results highlight the need for developing new interventions directed at reducing disability.

**Table:** 

**Key Points** • *Physical and mental disability is still considerable in contemporarily treated RA.* • *Interventions specifically aimed to reduce these disabilities need to be further developed.* • *Patients with severe disability need to be identified in clinical settings and offered person-centered rehabilitation.*

## Introduction

The practice of early diagnosis of rheumatoid arthritis (RA) and prompt start with disease-modifying antirheumatic drugs (DMARD), which was established during the late 1990s, was effective in reducing disease activity as well as disability [1–3]. In the early 2000s, further improvements in disease activity and disability were obtained with the widespread introduction of biological disease-modifying antirheumatic drugs (bDMARDs) [4–8]. Despite these improvements, some patients, especially women, continue to experience disability, a finding that suggests better interventions are needed [6, 9–12]. Physical disabilities are reported in long-term follow-up studies, but less is known about mental disabilities in contemporary “real-world” RA. Consequently, the main aim was to compare long-term follow-up of mental disabilities in RA patients diagnosed after the introduction of bDMARDs and treated according to contemporary national guidelines to patients diagnosed in the late 1990s. We also analyzed the relation between physical and mental disabilities.

## Materials and methods

### Patients

This study was based on two cohorts of patients with recent-onset RA (≤ 1-year symptom duration) included in the prospective Swedish multicenter project “Early Intervention in RA” (TIRA), aiming to establish routines for early diagnosis and multi-professional interventions.

The historic TIRA-1 cohort included 320 patients between 1996 and 1999 fulfilling either ≥ 4 of 7 ACR 1987 classification criteria (95%) or morning stiffness ≥ 60 min, symmetrical arthritis, and small joint arthritis (5%) [2]. Ten years later (2006–2009), after the widespread introduction of bDMARDs and treat-to-target strategies in clinical practice, 463 patients were included in the TIRA-2 cohort based on identical inclusion criteria as in TIRA-1 [8]. In both cohorts, patients were offered pharmacological treatment according to national guidelines, physiotherapy, occupational therapy, and social counselling based on individual needs at scheduled yearly follow-up visits up to 8 years after inclusion. In addition, patients were offered education 1 year after diagnosis and inclusion. Data on disease activity, DMARDs, and disabilities were registered as a part of the clinical routine at all visits.

### Medication and disease activity

Prescription of conventional synthetic DMARDs (csDMARDs) and biological DMARDs (bDMARDs) were registered at all visits. Disease activity was calculated using the 28-joint Disease Activity Score (DAS28) [13].

### Patient-reported physical and mental disability

Physical disability was assessed with Swedish versions of the Health Assessment Questionnaire (HAQ, score 0–3) [14] and the four physical scales in the Swedish version of the Short Form Health Survey (SF-36): Physical Functioning (PF), Role Physical (RP), Bodily Pain (BP), and Vitality (VT). The SF-36 also includes four aspects of mental health: General Health (GH), Social Functioning (SF), Role Emotional (RE), and Mental Health (MH). Each SF-36 scale is scored from 0 to 100, where 100 corresponds to full health [15].

### Statistics

Cross-sectional analyses were performed within and between the two cohorts based on the available data gathered at each timepoint. Descriptive statistics are presented as mean and standard deviation, and the independent samples *t*-test was used to test differences between groups. Pearson’s chi-square test was used to analyze differences in the distribution of DMARDs between groups. SPSS version 28 was used, and statistical significance was set at two-sided *p* < 0.05.

### Ethics

This study was performed in accordance with the ethical standards in the Declaration of Helsinki and its later amendments. All participants gave their informed consent before their inclusion in the study, and the protocol was approved by the local ethics committees associated with the participating rheumatologic units in Sweden: TIRA-1 (Dnr 96035) and TIRA-2 (Dnr M168-05).

## Results

### Patient characteristics

There were no significant differences at inclusion for age, DAS-28, rheumatoid factor (RF), and anti-citrullinated protein antibodies (ACPA) between cohorts, but women had higher HAQ scores in the TIRA-2 cohort than in the TIRA-1 cohort. TIRA-1 patients who were lost during follow-up were significantly older than the patients who attended the 8-year follow-up, but no significant differences were apparent at inclusion regarding sex distribution, DAS-28, HAQ score, RF, or ACPA. TIRA-2 patients who did not complete the 8-year follow-up were significantly older and more often seropositive than the remaining patients (Table 1).

**Table 1:** Differences at inclusion between cohorts stratified for sex

	Women			Men		
	TIRA-1	TIRA-2	*p*	TIRA-1	TIRA-2	*p*
Sex, number (%)	214 (67)	306 (67)		106 (33)	154 (33)	
Age, year (SD)	55 (16)	57 (15)	0.061	59 (15)	62 (13)	0.096
DAS-28, score (SD)	5.2 (1.2)	5.1 (1.2)	0.306	5.3 (1.1)	5.0 (1.3)	0.189
RF positive, number (%)	131 (62)	182 (59)	0.508	61 (58)	92 (62)	0.562
ACPA positive, number (%)	104 (63)	202 (70)	0.173	52 (68)	89 (64)	0.558
HAQ, score (SD)	0.92 (0.60)	1.05 (0.60)	0.014	0.81 (0.53)	0.89 (0.62)	0.309

*TIRA* Early Intervention in Rheumatoid Arthritis, *DAS-28* Disease Activity Score 28-joint count, *RF* rheumatoid factor, *anti-CCP* anti-citrullinated protein, *HAQ* Health Assessment Questionnaire

### Comparison between cohorts

**Fig. 1:**
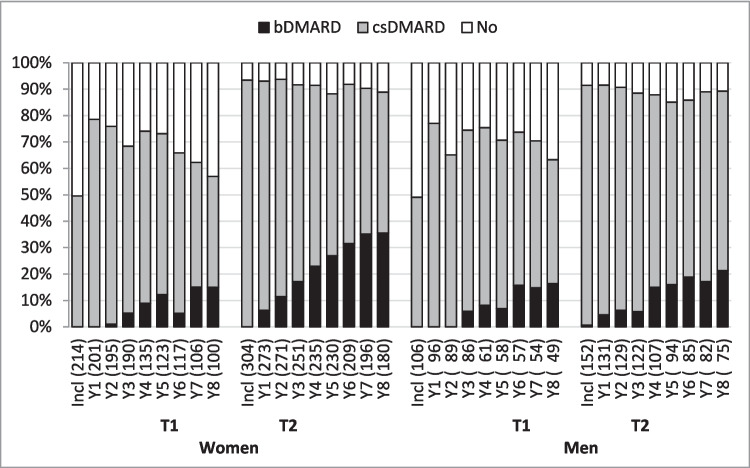
Percentages of prescribed conventional synthetic disease-modifying antirheumatic drugs (csDMARDs) and biologic disease-modifying antirheumatic drugs (bDMARDs) at inclusion (Incl) and yearly (Y) follow-ups in TIRA-1 (T1) cohort and TIRA-2 (T2)

**Fig. 2:**
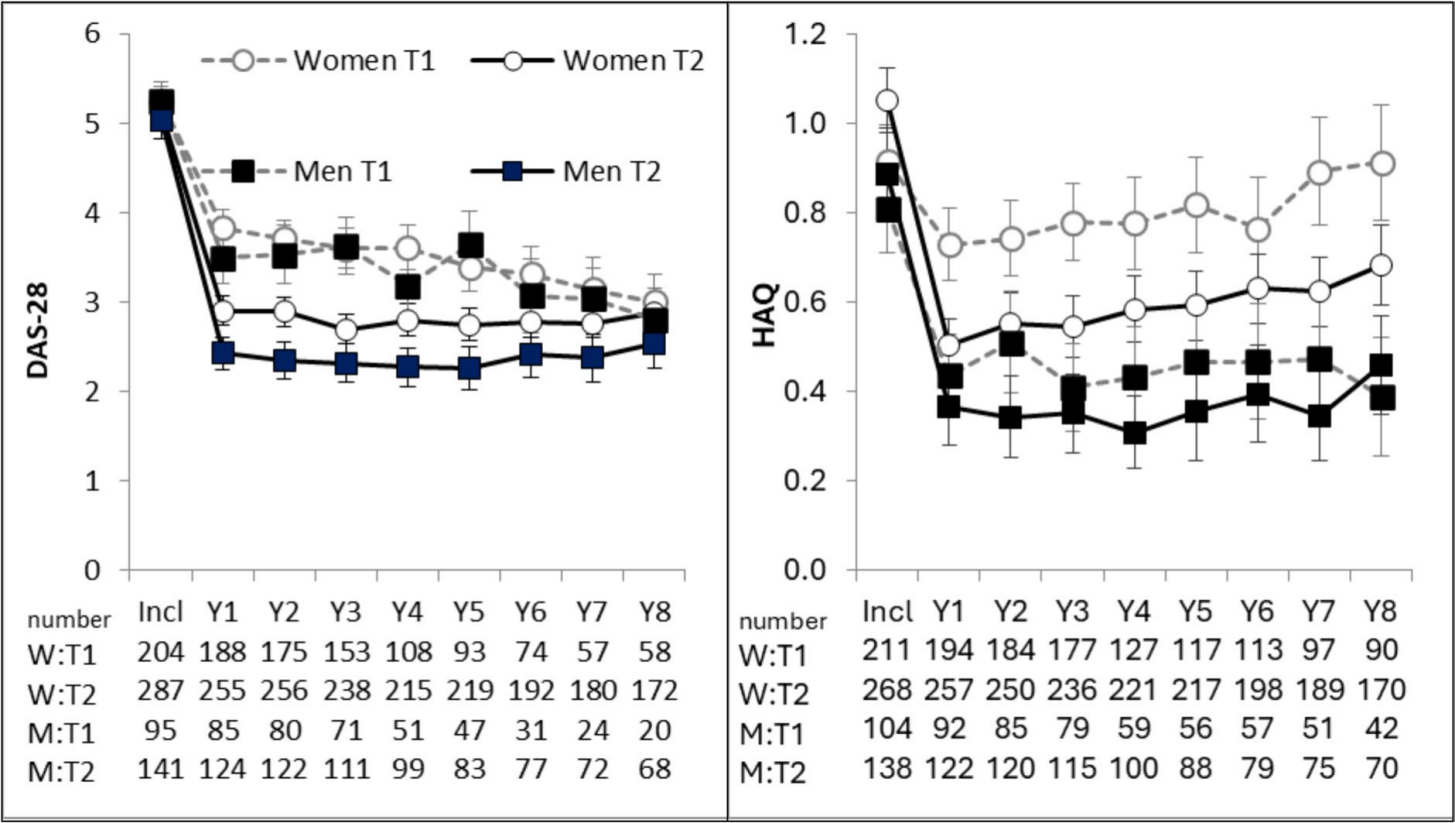
Means and SD for Disease Activity Score 28-joint count (DAS-28) and physical disability reported with Health Assessment Questionnaire (HAQ) in women and men at inclusion (Incl) and at the yearly (Y) follow-ups in TIRA-1 (T1) cohort and in the TIRA-2 (T2) cohort

Significantly more women were prescribed DMARDs at all visits in the TIRA-2 cohort than in the TIRA-1 cohort (*p* < 0.001). Similarly, significantly more men were prescribed DMARDs at all visits in the TIRA-2 cohort than in the TIRA-1 cohort (*p* < 0.05) except for year 6 (Fig. 1). Disease activity did not differ between cohorts at inclusion, but DAS-28 was significantly lower in TIRA-2 than in TIRA-1 at most of the yearly follow-ups (*p* < 0.001), except for year 7 (*p* = 0.06) and year 8 (*p* = 0.52) for women, and for men at year 8 (*p* = 0.34) (Fig. 2). At inclusion, women had more physical disability according to HAQ in TIRA-2 compared to the TIRA-1 cohort (Fig. 2). For men, the only significant difference was lower HAQ in the TIRA-2 cohort at year 2 (Fig. 2).

**Fig. 3:**
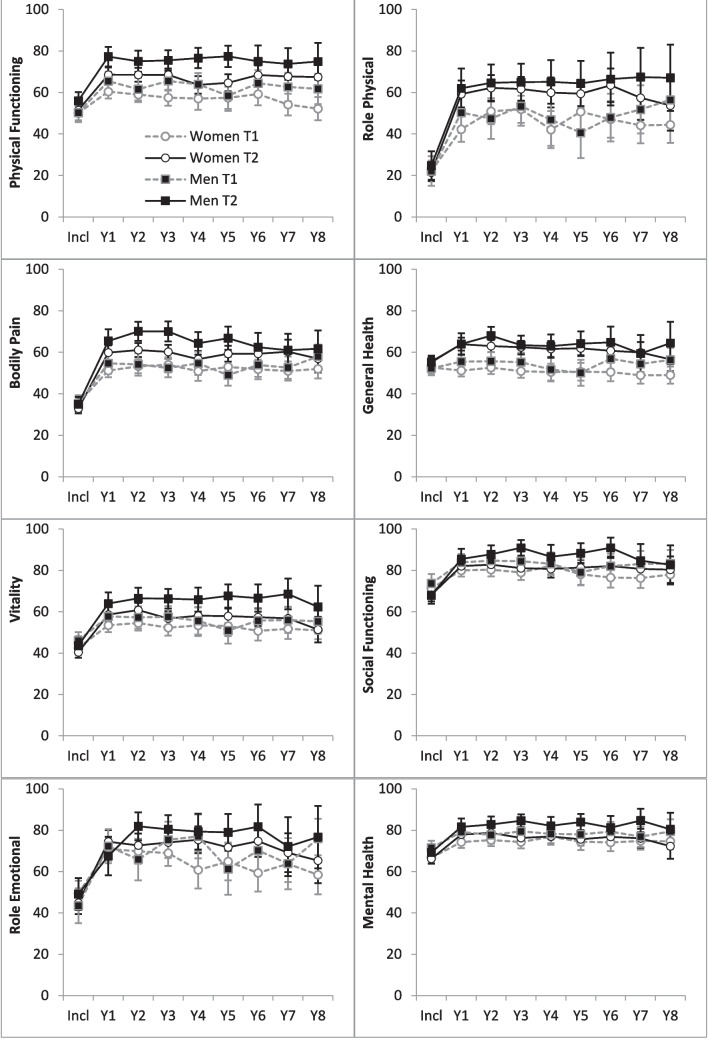
Means and SD for patient-reported physical and mental disability with Short Form 36 (SF-36) in women and men at inclusion (Incl) and at the yearly (Y) follow-ups in the TIRA-1 cohort (T1) and TIRA-2 cohort (T2)

Although similar at inclusion, women in the TIRA-2 cohort reported significantly lower physical and mental disability for many of the SF-36 scales and at most follow-ups than women in the TIRA-1 cohort at year 2 (Fig. 3). The exceptions were the two mental SF-36 scales Mental Health and Social Functioning, which showed no significant differences between cohorts. Men reported significantly lower disability in most SF-36 scales in the TIRA-2 cohort than in the TIRA-1 cohort.

**Fig. 4:**
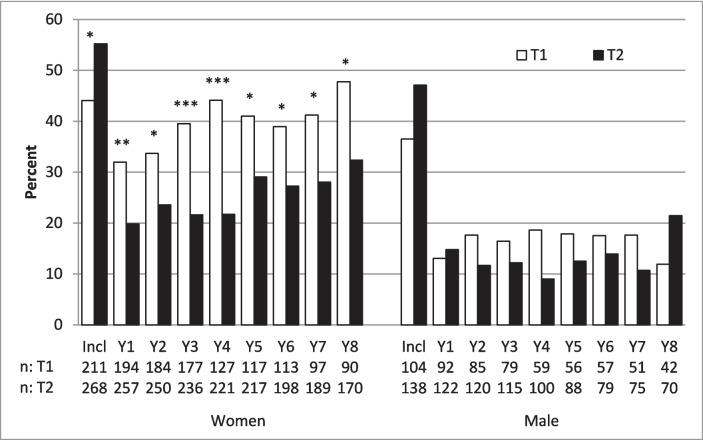
Percentage of women and men within subgroups of patients with a Health Assessment Questionnaire (HAQ) score ≥ 1 at inclusion (Incl) and at yearly follow-ups in the TIRA-1 cohort (T1) and TIRA-2 cohort (T2)

We stratified patients based on HAQ score ≥ 1, the level suggested to define substantial disability [2]. A significantly lower proportion of women reported an HAQ score ≥ 1 at the 8-year follow-up in the TIRA-2 cohort than in the TIRA-1 cohort (32% vs. 48%, *p* = 0.015). The corresponding analysis in men did not reach statistical significance (21% vs. 12%; *p* = 0.203) (Fig. 4).

### Comparison between sexes in TIRA-2

At inclusion, men were significantly older than women (62 vs. 57 years, *p* < 0.01). In the TIRA-2 cohort, significantly more women were prescribed DMARDs than men except at years 3, 6, and 7 (Fig. 1). Disease activity did not differ significantly at inclusion or at the 8-year follow-up but was significantly higher among women at all other visits (Fig. 2).

At all visits, women reported significantly more physical disability than men according to the HAQ. There were no significant differences between sexes at inclusion for the SF-36 physical and mental scales. At follow-up, there were no significant sex differences for General Health, Role Emotional, and Role Physical, but men trended towards better status than women in Mental Health, Physical Functioning, and Social Functioning at many follow-ups (Fig. 3).

### HAQ score related to SF-36 at year 8 in TIRA-2 cohort

Women with HAQ ≥ 1 at year 8 (32%) compared to women with HAQ < 1 at year 8 reported more physical and mental disabilities in all SF-36 scales except Mental Health. The 21% of men who had HAQ ≥ 1 reported significantly more disability in three SF-36 scales: Bodily Pain, Physical Functioning, and Role Physical. The only SF-36 scale that was not significantly different between sexes was Mental Health (Table 2).

**Table 2:** Means and SD for DAS-28 and the scales in SF-36 within subgroups of patients with HAQ score ≥ 1 and < 1 at year 8 in TIRA-2 cohort

	Women			Men		
	HAQ ≥ 1, *M* (SD)	HAQ < 1, *M* (SD)	*p*	HAQ ≥ 1, *M* (SD)	HAQ < 1, *M* (SD)	*p*
DAS-28 (score)	3.56 (1.16)	2.50 (1.11)	** < 0.0001**	3.61 (1.02)	2.28 (0.93)	** < 0.0001**
Physical Functioning	35 (19)	70 (21)	** < 0.0001**	43 (22)	70 (21)	**0.003**
Role Physical	31 (34)	56 (42)	** < 0.0001**	25 (16)	63 (34)	**0.001**
Bodily Pain	43 (18)	61 (23)	** < 0.0001**	39 (11)	62 (17)	**0.001**
General Health	43 (15)	56 (20)	** < 0.0001**	49 (30)	61 (16)	0.376
Mental Health	69 (20)	76 (18)	0.071	66 (28)	82 (15)	0.181
Role Emotional	38 (43)	73 (36)	** < 0.0001**	67 (27)	78 (30)	0.340
Social Functioning	70 (24)	85 (21)	**0.001**	63 (31)	85 (17)	0.105
Vitality	43 (18)	56 (20)	** < 0.0001**	55 (25)	58 (19)	0.736

*DAS-28* Disease Activity Score 28-joint count, *SF-36* Short Form 36, *HAQ* Health Assessment Questionnaire

## Discussion

In this long-term follow-up of well-characterized patients, we find that contemporary management of early RA is associated with a more substantial initial improvement in disability and disease activity compared to the historic cohort. We also found that this more favorable disease course persisted over 8 years. Despite these improvements, patient-reported disability related to RA remained a significant problem in the TIRA-2 cohort. This is illustrated by the fact that 32% of the women that had HAQ score ≥ 1 after 8 years also reported substantial physical and mental disability by SF-36. The corresponding percentage in men was 12%.

At time of inclusion, no significant differences between the cohorts were evident regarding disease activity or SF-36. However, a slightly higher mean age was found in the contemporary treated TIRA-2 cohort than in the TIRA-1 cohort, which could explain the higher HAQ score in women in the TIRA-2 cohort [16]. Independent of these differences, the course was not less favorable in the TIRA-2 cohort but rather the opposite. As expected, DMARD therapy was more intense both at inclusion and throughout the follow-ups in the TIRA-2 cohort than in the TIRA-1 cohort, a finding that tracks with changes in treatment guidelines [1].

The initial reduction of physical disability in both TIRA cohorts and the higher HAQ scores in women than in men correspond with previous findings [5–7]. Also, less physical disability during follow-up in our contemporarily treated TIRA-2 cohort compared to our historic TIRA-1 cohort confirms earlier findings [5, 7]. Despite these improvements, the HAQ scores reflect considerable physical disability in the TIRA-2 cohort at year 8 compared to Swedish referents (women 0.07; men 0.05) [17]. In addition, the physical scale mean scores in the SF-36 for the TIRA-2 show worse health compared to norms in the Swedish population, and this is most pronounced in women [18].

Although the SF scales Role Physical and General Health in the TIRA-2 cohort revealed improved health, these scores show considerably lower health than for the Swedish norms in the Swedish population which was most pronounced for women [18]. However, the SF-36 scales Social Functioning and Mental Health showed no differences between cohorts at inclusion and no differences between sex. And after the initial improvement, these SF-36 scores were in line with Swedish norms [18]. To the best of our knowledge, there are no other long-term follow-up studies of the SF-36 scales for a cohort similar to the TIRA-2 cohort, which could be used for comparisons. Our analysis of the eight individual SF-36 scales adds new information to the field. However, the alternative method to calculate two aggregated SF-36 scores—one for the four physical scales (PSC) and one for the four mental scales (MSC)—has been used in other early RA follow-up studies. A recently published review reports that the PSC score, after an initial improvement, shows better health during the first 5 years in patients diagnosed after 2002 compared to those diagnosed earlier [7]. Despite not being stratified by sex, these improvements are in line with the improvements in the separate physical scales for women and men in the TIRA-2 cohort. The MSC score that is stable over the follow-ups shows no differences between the pre- and post-2002 patients, a finding that is in line with the mental SF-36 scales Social Functioning and Mental Health but differs from the improvement in General Health and Role Physical in the TIRA-2 cohort for both women and men. In the ESPOIR cohort, which includes RA patients diagnosed in the early 2000s, MSC and PSC scales show an improvement 10 years after diagnosis compared to the time of diagnosis, although sex differences were not analyzed [5]. Our results for the SF-36 scales over 8 years, separately, that show sex differences, improvement in six of the SF-36 scales, and discriminating outcomes between the mental SF-36 scales have, to the best of our knowledge, not been reported earlier.

Today’s knowledge about physical and mental disabilities and unmet needs for patients suffering with RA reveals several challenges [9, 11, 19]. There is a need for a forthcoming implementation of available effective non-pharmacologic and person-centered rehabilitation programs to achieve additional improvements [6, 12, 20–22]. Person-centered interventions can be initiated already in primary care after diagnosis of RA [23]. Patients with such needs can probably be identified using critical levels for self-reported disability such as HAQ score or pain intensity [2, 24], but more well-designed studies are required to evaluate the effectiveness of available interventions in such settings [7, 25, 26]. Goalsetting for self-management has been proposed as a part of rehabilitation to obtain self-efficacy and reduce anxiety and depression [27]. Available long-term follow-up studies of disability with quantitative methods in well-treated contemporary early RA patients point out a further need for developing new strategies, including preventive interventions, directed at disability as such [1–10]. In addition, interview studies of patients and their significant others suggest further requirements for the healthcare system to develop methods and routines that involve significant others early in the rehabilitation process [28].

In this study, we shed light on sex-stratified disability in contemporarily treated RA with the eight scales in SF-36 and compared this to historical data. To the best of our knowledge, this has not been reported earlier. Still, more knowledge is needed in the area of RA and rehabilitation, such as the identification of early risk factors for extensive disability and the development of novel rehabilitation strategies.

## Conclusions

Despite modern multi-professional management of RA designed according to national guidelines, physical and mental disability remains considerable in RA patients, especially among women. Our results highlight the need for person-centered non-pharmacological rehabilitation programs that optimize both physical and mental function and participation in daily life for patients with RA. Further implementation of available effective non-pharmacologic and person-centered rehabilitation programs is needed together with further development of interventions directed to reduce both the physical and mental disability associated with RA itself.

## Data Availability

From the authors upon reasonable request.
